# Effects of Ultrasonic Nanocrystal Surface Modification on the Formation of a Nitride Layer in Ti-6Al-4V Alloy

**DOI:** 10.3390/ma18153487

**Published:** 2025-07-24

**Authors:** Bauyrzhan Rakhadilov, Nurtoleu Magazov, Zarina Aringozhina, Gulzhaz Uazyrkhanova, Zhuldyz Uazyrkhanova, Auezhan Amanov

**Affiliations:** 1 PlasmaScience LLP, Ust-Kamenogorsk 070000, Kazakhstan; rakhadilovb@gmail.com; 2 D. Serikbayev East Kazakhstan Technical University, 69 Protozanov Street, Ust-Kamenogorsk 070000, Kazakhstan; magazovn@gmail.com (N.M.); zaringozhina@edu.ektu.kz (Z.A.); zhuazirhanova@edu.ektu.kz (Z.U.); 3 Faculty of Engineering and Natural Sciences, Tampere University, FIN-33014 Tampere, Finland

**Keywords:** Ti-6Al-4V, elastic modulus, hardness, coefficient of friction, ion-plasma nitriding, UNSM

## Abstract

This study investigates the effects of ultrasonic nanocrystalline surface modification (UNSM) on the formation of nitride layers in Ti-6Al-4V alloy during ion-plasma nitriding (IPN). Various UNSM parameters, including vibration amplitude, static load, and processing temperature, were systematically varied to evaluate their influence on microstructure, hardness, elastic modulus, and tribological behavior. The results reveal that pre-treatment with optimized UNSM conditions significantly enhances nitrogen diffusion, leading to the formation of dense and uniform TiN/Ti_2_N layers. Samples pre-treated under high-load and elevated-temperature UNSM exhibited the greatest improvements in surface hardness (up to 25%), elastic modulus (up to 18%), and wear resistance, with a reduced and stabilized friction coefficient (~0.55). Scanning electron microscopy (SEM) and X-ray diffraction (XRD) analyses confirmed microstructural densification, grain refinement, and increased nitride phase intensity. These findings demonstrate not only the scientific relevance but also the practical potential of UNSM as an effective surface activation technique. The hybrid UNSM + IPN approach may serve as a promising method for extending the service life of load-bearing biomedical implants and engineering components subjected to intensive wear.

## 1. Introduction

Recent advances in materials science are focused on the development of innovative strengthening technologies for structural materials that are in high demand in the aerospace, medical, and automotive industries [1]. Particular attention is given to surface treatment methods aimed at improving the operational properties of materials, such as wear resistance, hardness, and corrosion resistance. Among various structural materials, the titanium alloy Ti-6Al-4V holds a leading position due to its high specific strength, low density, biocompatibility, and resistance to aggressive environments [2]. However, its relatively low surface hardness limits the alloy’s application under conditions of intensive friction and wear [3].

To overcome these limitations, surface engineering techniques have been intensively explored. Among them, ion-plasma nitriding (IPN) is widely adopted to improve surface properties by forming hard titanium nitrides (TiN, Ti_2_N) on the alloy surface. These nitride phases exhibit high hardness (~1800–2000 HV), chemical stability, and improved wear and corrosion resistance [4,5]. Nevertheless, the depth and uniformity of the nitrided layer are often restricted by the presence of a passive oxide film, low surface defect density, and poor nitrogen diffusivity of titanium alloys [6]. These factors result in thin, brittle, or poorly adhered nitrided layers with limited mechanical performance [7].

In response, pre-activation of the surface has emerged as a promising approach to enhance nitrogen uptake during nitriding. One of the most advanced techniques in this context is ultrasonic nanocrystalline surface modification (UNSM), which creates a nanostructured layer with high dislocation density and refined grains through high-frequency mechanical impacts [8,9]. This surface architecture promotes nitrogen diffusion, leading to more uniform and thicker nitrided layers in the subsequent IPN step [10]. Moreover, it improves adhesion and reduces the likelihood of delamination or cracking during service.

Although prior studies have highlighted the benefits of combining UNSM and IPN, the literature still lacks systematic analyses of how different UNSM parameters—such as vibration amplitude, static load, and processing temperature—affect the structural evolution, phase composition, and tribo-mechanical performance of the nitrided layer. Additionally, comparisons with conventional surface treatments like shot peening, PVD coatings, and laser shock peening demonstrate that those methods offer limited capability to modify the surface grain structure or activate chemical diffusion [11,12,13,14,15,16,17,18,19,20,21,22,23,24,25,26,27]. Unlike them, UNSM enables precise parameter control and generates a dense, defect-rich surface that synergistically interacts with nitrogen plasma during IPN, improving the final performance.

To better understand the current state of research on the surface modification of Ti-6Al-4V alloys, a comparative summary of the recent studies is presented in Table 1. It outlines the various pre- and post-treatment approaches, their key processing parameters, and effects on surface hardness, elastic modulus, wear and corrosion resistance, and fatigue performance.

Furthermore, hybrid approaches such as UNSM-assisted ion-plasma nitriding are more energy-efficient than conventional high-temperature nitriding, offering sustainable, low-emission solutions for the surface enhancement of titanium-based components.

The motivation for the present study stems from the need to establish an optimized hybrid processing route that maximizes the effectiveness of ion-plasma nitriding through tailored UNSM conditions. Therefore, this work investigates the influence of pre-nitriding UNSM treatments under varying amplitudes, loads, and temperatures on the microstructure, mechanical properties, and tribological behavior of Ti-6Al-4V alloy. This study not only contributes to a deeper understanding of UNSM-assisted nitriding but also proposes an efficient pathway for designing high-performance surfaces for biomedical and aerospace applications.

## 2. Materials and Methods

### 2.1. Materials

The objective of this study is to evaluate the effect of UNSM on the formation of a nitride layer in Ti-6Al-4V alloy during ion-plasma nitriding. The chemical composition of the Ti-6Al-4V alloy is shown in Table 2. Special attention is given to the microstructure and tribo-mechanical properties, including hardness, elastic modulus, and wear resistance.

### 2.2. Research Methods

**Figure 1:**
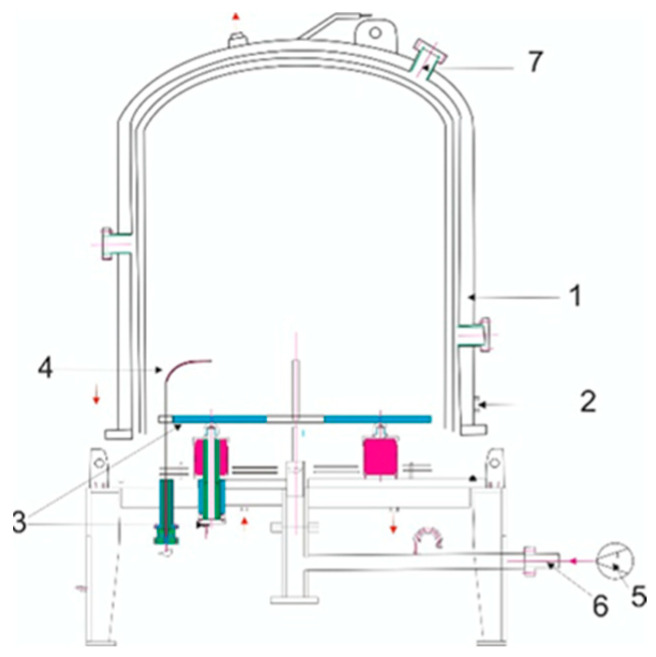
Schematic diagram of the ion nitriding equipment: 1—vacuum chamber; 2—anode; 3—cathode (specimen holder); 4—vacuum chamber temperature sensor; 5—pumping system; 6—pressure relief plug; 7—vacuum viewing window.

**Figure 2:**
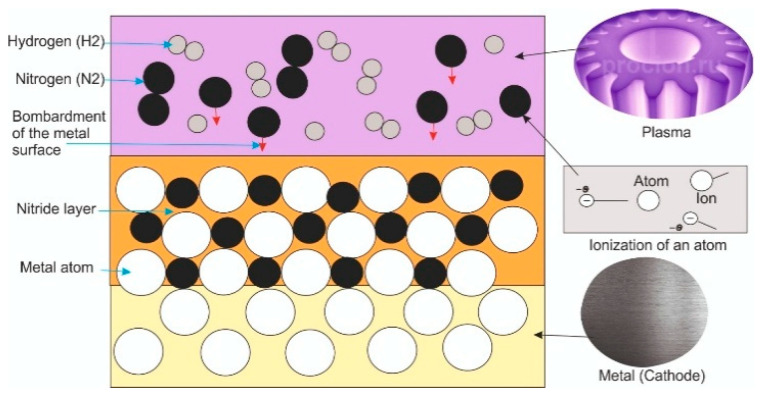
The process of gas ion diffusion into the metal surface.

The surfaces of the specimens were treated using UNSM. During the UNSM process, the following parameters were varied: amplitude, static load, and temperature (Table 3). The selected UNSM regimes were based on the results of our previous study [14], which showed that these specific parameters ensure effective grain refinement and an increase in both microhardness and elastic modulus of the Ti-6Al-4V alloy. After the treatment, the specimens were air-cooled to room temperature. The UNSM-treated surfaces were then subjected to ion-plasma nitriding (IPN). The IPN process was carried out using a specialized laboratory setup (Model LDMC-20, Tianman Industrial Furnace, Tianjin, China). A schematic representation of the equipment is shown in Figure 1. The treatment was performed at a temperature of 500 °C for 2 h. The pressure in the chamber was maintained at 400 Pa. A glow discharge was initiated between the anode (the vacuum chamber body) and the cathode (the metal specimen) upon application of voltage. This discharge ionizes nitrogen atoms by knocking out electrons, forming nitrogen ions. These ions bombard the metal surface and penetrate into it, leading to the formation of a nitride lattice (Figure 2).

To evaluate the tribo-mechanical characteristics of the specimens, their microhardness, elastic modulus, and tribological properties were investigated. Hardness measurements were performed using a Vickers indenter on a FISCHERSCOPE HM2000S device (Helmut Fischer GmbH, Sindelfingen, Germany). The indenter was a four-sided diamond pyramid with an angle of 136° between the opposite faces. Hardness was determined according to the Vickers scale (HV). The elastic modulus was calculated using the instrumented indentation method on the same device. All measurements were conducted according to the Vickers scale (HV), and each test was repeated five times to ensure accuracy. Tribological properties were evaluated in accordance with the ASTM G99 standard using a TRB^3^ tribometer (Anton Paar GmbH, Graz, Austria). A ball-on-disc configuration was used, where a 6.00 mm diameter 100Cr6 steel ball served as the counter body. The tests were conducted in ambient air at a laboratory temperature of 21.33 °C and relative humidity of 31.05%. The sliding radius was 2.00 mm, and the linear sliding speed was set to 10.0 cm/s. A constant normal load of 2.00 N was applied, and data were recorded at an acquisition rate of 10 Hz. The total sliding distance was 300.00 m. The test was conducted in a single-pass mode without pause, with initial homing enabled and no unloading at the end. The Ti-6Al-4V specimens were used as substrates without additional cleaning prior to testing. The phase composition was analyzed using an X’Pert Pro X-ray diffractometer (Panalytical, Amsterdam, The Netherlands) with Cu-Kα radiation, operating at 40 kV and 30 mA. The scanning parameters were set to 35° < 2θ < 85°, with a step size of 0.02° and an exposure time of 5 s. The microstructure of the specimens was analyzed using a scanning electron microscope (TESCAN Vega, Tescan, Brno, Czech Republic). Prior to examination, cross-sectional samples were prepared through a series of standard metallographic procedures. First, the specimens were mechanically sectioned using silicon carbide cutting wheels. After sectioning, the surfaces were sequentially ground and polished using diamond paste to achieve a flat and deformation-free finish. To reveal the microstructural features, the polished samples were chemically etched for 10 s using Kroll’s reagent, which consists of 100 mL distilled water, 1–3 mL hydrofluoric acid, and 2–3 mL nitric acid.

## 3. Research Results and Discussion

### 3.1. Tribology Evaluation Results

**Figure 3:**
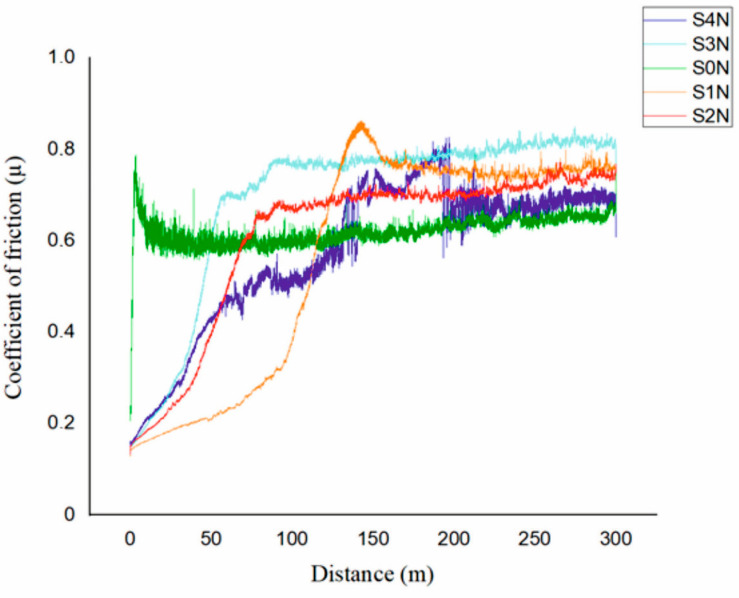
Results of tribological tests under various UNSM treatment parameters after nitriding.

**Figure 4:**
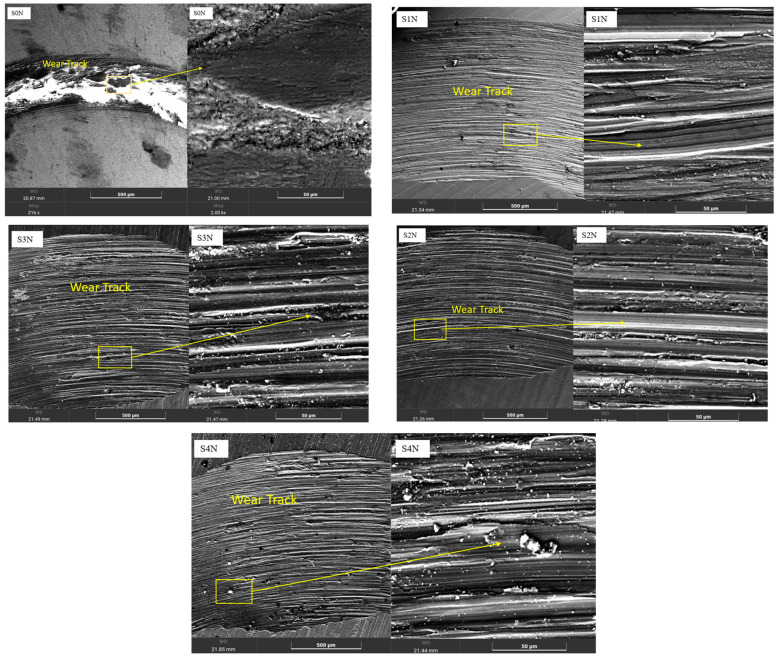
SEM images of the wear surfaces of Ti-6Al-4V specimens after combined treatment by UNSM and nitriding.

Based on the analysis of the coefficient of friction (Figure 3) and the wear surface morphology (Figure 4), it was established that the parameters of UNSM significantly affect the tribological performance of the nitrided Ti-6Al-4V alloy.

All samples exhibited two distinct stages: a running-in phase, during which the coefficient of friction (μ) increased rapidly, followed by a steady-state phase with stabilized friction behavior. The S4N sample (amplitude of 30 μm, load of 60 N, temperature of 400 °C) showed the lowest and most stable friction coefficient (~0.55) after a short running-in period (~40 m). SEM analysis revealed a uniform and dense wear track with minimal signs of plastic deformation, indicating high wear resistance and the presence of a protective nitride layer. The dominant wear mechanism is identified as mild abrasive wear. The S3N sample exhibited a slightly longer running-in phase and a steady-state of ~0.60–0.65. SEM images showed localized craters, micro-scratches, and indications of fatigue wear. Although the nitride layer was formed, its local heterogeneity led to selective degradation during prolonged contact. The S2N and S1N samples, treated at room temperature, displayed less stable friction behavior and a prolonged running-in period. Their coefficients of friction reached up to ~0.75 and exhibited fluctuations. SEM images revealed pronounced grooves, microcracks, and delamination zones. The prevailing wear mechanisms in these samples were abrasive and delamination wear, resulting from weak adhesion and insufficient depth of the nitrided layer. The S0N reference sample, without UNSM treatment, exhibited the most unstable friction behavior. After a short running-in phase, the coefficient of friction rapidly rose to ~0.80 and showed sharp fluctuations. SEM analysis indicated severe surface degradation, including plastic deformation and material detachment, consistent with intensive adhesive wear due to the lack of a reinforced surface layer. In summary, pre-treatment UNSM with optimized parameters enhances the formation of a dense and uniform nitrided layer, significantly improving wear resistance and stabilizing the frictional response. The S4N mode, in particular, proved to be the most effective, delivering superior tribological performance due to the combined effect of high amplitude, load, and temperature. In comparison to laser shock-peened and PVD-coated Ti-6Al-4V alloys, which typically exhibit coefficients of friction in the range of 0.65–0.80 under similar dry sliding conditions, our samples achieved more stable friction behavior and lower steady-state values (e.g., 0.55 for S4N), highlighting the tribological advantage of the combined UNSM and IPN approach [15].

### 3.2. Results of SEM (Scanning Electron Microscopy)

**Figure 5:**
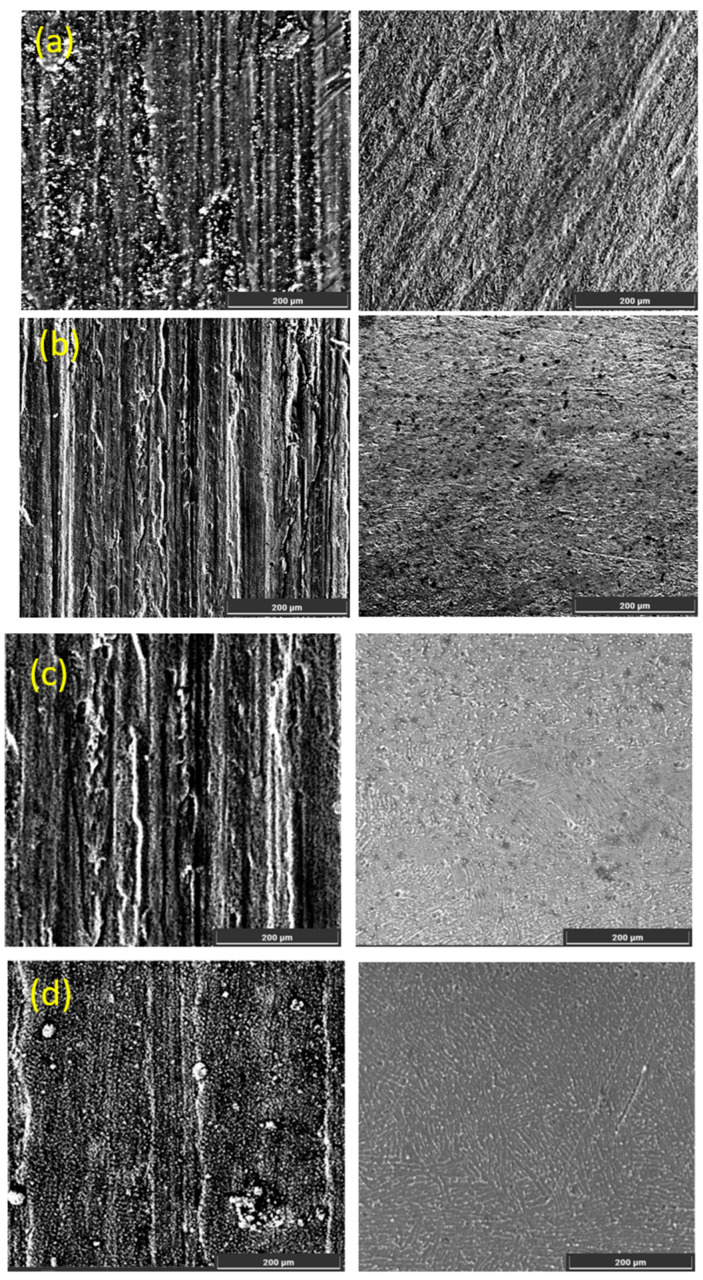
SEM images of the Ti-6Al-4V alloy surface after nitriding with various UNSM parameters: S1N (**a**); S2N (**b**); S3N (**c**); and S4N (**d**).

Figure 5 shows the SEM images of the Ti-6Al-4V alloy surface after IPN with various UNSM parameters. In the S1N sample (amplitude of 20 μm, load of 30 N, RT), the surface structure remains relatively smooth and weakly granular. The absence of pronounced surface deformation indicates a limited activation of diffusion processes and a low density of lattice defects, which hinders the effective formation of the nitrided zone [16]. Under the S2N mode (amplitude of 30 μm, load of 30 N, RT), the surface exhibits a more pronounced deformed relief and enhanced grain refinement, though the structure remains locally heterogeneous. This may lead to uneven nitrogen saturation, reducing the stability and uniformity of the nitride layer [17]. The S3N sample (amplitude of 30 μm, load of 50 N, temperature of 400 °C) demonstrates a uniformly deformed and densified surface with clear signs of compaction. The elevated UNSM temperature promotes diffusion activation, substructural zoning, and accelerated nucleation of nitride phases, providing favorable conditions for the formation of a dense diffusion layer [18]. The most pronounced structural densification was observed in the S4N sample (amplitude of 30 μm, load of 60 N, temperature of 400 °C). The surface exhibited a dense nanostructured zone with uniform grain refinement and no microcracks. Such a condition ensures the highest nitriding efficiency by promoting the formation of a strong and stable nitride layer, as reported in previous studies [18]. These structural analysis results confirm that preliminary ultrasonic surface modification generates an activated substrate, which enhances nitrogen diffusion and enables the controlled formation of a hardened nitrided layer. The higher the mechanical and thermal energy applied during pre-treatment, the more effective the structural preparation of the surface. Incorporating evidence from the literature, Liu et al. reported that UNSM-treated Ti-6Al-4V alloy formed nitride layers with thicknesses of approximately 0.26 µm and 1.35 µm after gas nitriding at 700 °C and 800 °C, respectively Accordingly, in the present work, the estimated thickness of the hardened nitride layer is expected to be in the range of 0.3 to 1.4 µm, depending on the UNSM parameters applied [16]. Moreover, the formation of a dense, nanostructured nitride layer observed in this study aligns with trends reported in prior work on UNSM-assisted diffusion processes but offers improved phase uniformity compared to conventional shot peening pretreatments [19].

### 3.3. X-Ray Phase Analysis

**Figure 6:**
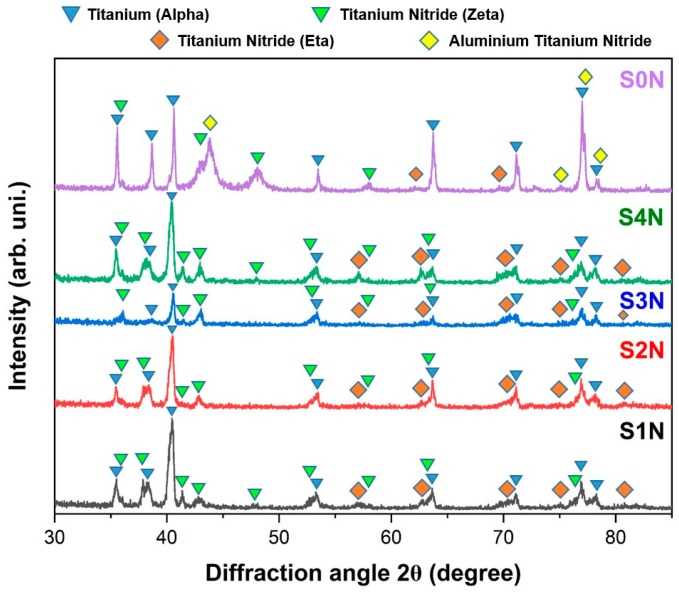
X-ray diffraction (XRD) patterns of Ti-6Al-4V alloy after IPN with various UNSM treatment parameters.

Figure 6 presents the XRD patterns of Ti-6Al-4V specimens subjected to ion-plasma nitriding after UNSM treatment with varying parameters, as well as a reference sample S0N that was nitrided without prior ultrasonic treatment. In the S0N sample, prominent peaks of the α-Ti matrix dominate the pattern, accompanied by weak reflections corresponding to the AlTi_3_N and Ti_2_N phases, indicating a relatively thin and less developed nitride layer. In contrast, the S1N sample (processed with minimum UNSM parameters) also shows dominant α-Ti peaks and only minor TiN signals, confirming limited nitrogen diffusion. The S2N pattern demonstrates a slight increase in the TiN peak intensity due to enhanced plastic deformation; however, the formation of the nitride layer remains insufficient without applying an elevated temperature. A significant increase in TiN peak intensity is observed in the S3N sample, alongside broader peaks that indicate a denser and more refined nitride layer. This result is attributed to the higher static load and processing temperature during UNSM. The S4N sample exhibits the highest intensity and sharpness of TiN and Ti_2_N peaks, suggesting the formation of a thick, uniform, and nanocrystalline nitride layer. These findings confirm that pre-treatment by UNSM plays a crucial role in promoting phase transformations during nitriding. The combination of higher mechanical impact and thermal energy during UNSM, particularly in the S4N mode, provides optimal conditions for the TiN phase formation, as also supported by previous studies [9]. Thus, the XRD results confirm that preliminary ultrasonic surface modification plays a decisive role in phase transformations during ion-plasma nitriding, providing favorable conditions for the intensive formation of TiN phases. The most pronounced effect is achieved when high temperature and static load are combined during the UNSM process, as demonstrated in the S4N mode.

The crystallite size (D) of the sample was calculated using the Scherrer equation:

D = Kλ/(β · cosθ)(1)

where K = 0.9 is the shape factor; λ = 0.15406 nm is the wavelength of Cu Kα radiation; β is the full width at half maximum (FWHM) of the diffraction peak (in radians); and θ is the Bragg angle.

The average crystallite size was determined to be approximately 8.07 nm, indicating the formation of a fine-grained nanostructure.

In addition, the microstrain (ε) in the crystal lattice was evaluated using the following relation:

ε = β/(4 · tanθ)(2)

The calculated average microstrain was approximately 0.0246 (2.46%), suggesting the presence of internal lattice distortions and structural defects induced by surface treatment.

### 3.4. Hardness

**Figure 7:**
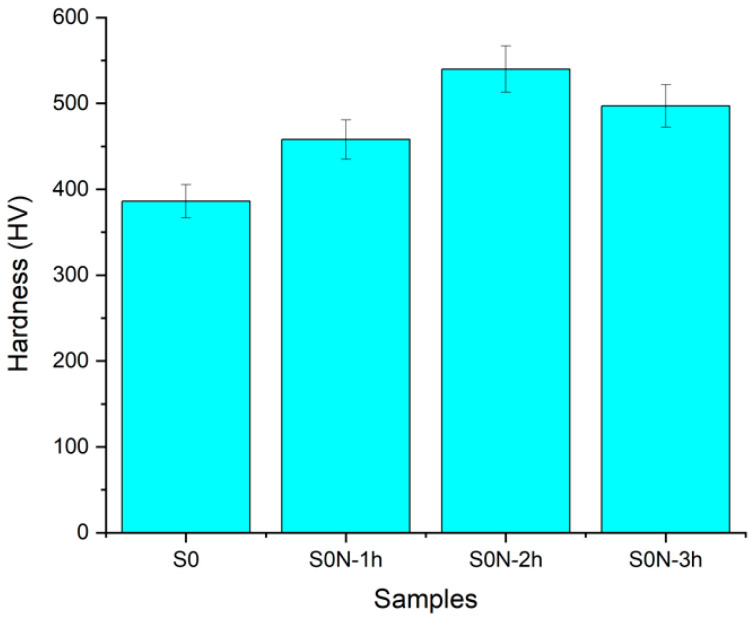
Variation in the hardness of Ti-6Al-4V specimens at 500 °C with different nitriding durations.

The parameters of the ion-plasma nitriding process were selected based on experimental data, which showed that the maximum surface microhardness of the Ti-6Al-4V alloy was achieved at a temperature of 500 °C and a duration of 2 h (Figure 7). When the treatment time was increased to 3 h, a decrease in hardness was observed, which was likely associated with grain coarsening, possible recrystallization, relaxation of internal stresses, the formation of defects (such as cracks and delamination), and the homogenization of the nitrogen concentration gradient due to extended diffusion saturation. Similar effects were reported by Minoru Umemoto [13], where excessive nitriding time resulted in a reduction in hardness due to grain growth and the release of residual stresses. Therefore, the temperature of 500 °C provides active nitrogen diffusion without structural degradation of the material, making it optimal for the treatment of the Ti-6Al-4V alloy.

**Figure 8:**
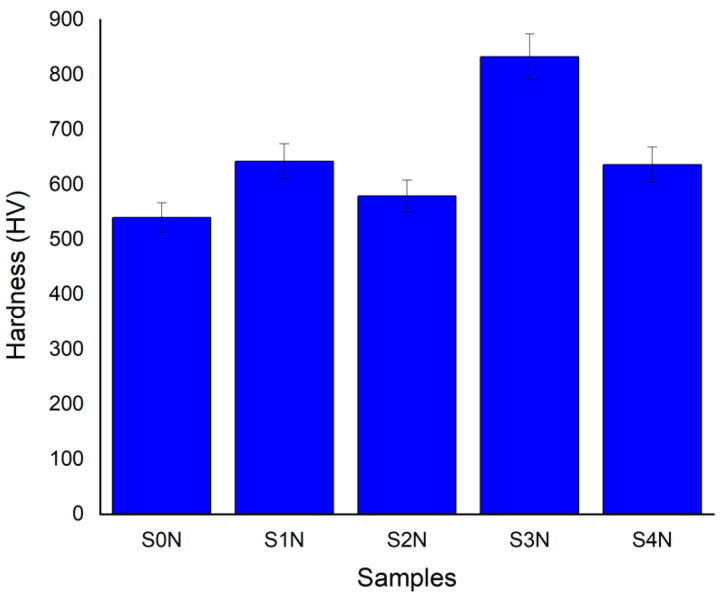
Effect of different UNSM regimes on the hardness of Ti-6Al-4V alloy after nitriding.

The results presented in Figure 8 clearly demonstrate the pronounced effect of the combination of UNSM and subsequent nitriding on the enhancement of surface hardness. Specifically, the combined treatment under the S1N regime resulted in more than a 25% increase in microhardness compared to the specimen subjected to nitriding alone (S0N). This indicates that preliminary UNSM treatment facilitates the active diffusion of nitrogen atoms into the material and the formation of hard nitride phases [20]. Such an enhancement is consistent with the findings of Sun et al. [21] and Mogucheva [22], which emphasize the significant role of prior plastic deformation in improving the conditions for nitrogen diffusion and the formation of the nitride layer. The S2N specimen, processed under similar conditions but with an increased amplitude (30 μm), exhibited a 17% increase in microhardness, further confirming the effectiveness of more intense surface deformation.

In the S3N regime, at a temperature of 400 °C, the microhardness increased by 23%, confirming the activation of nitrogen diffusion processes due to enhanced atomic mobility. This effect has been reported in several studies, which have also emphasized the role of thermal activation in increasing the thickness and density of the nitride layer [23]. The least pronounced effect was observed for the S4N specimen, where UNSM was performed at 400 °C with a load of 60 N. The increase in microhardness was only 9.6%, which may be attributed to partial recrystallization, a reduction in defect density, or local overheating that diminished the plastic deformation effect [13].

**Figure 9:**
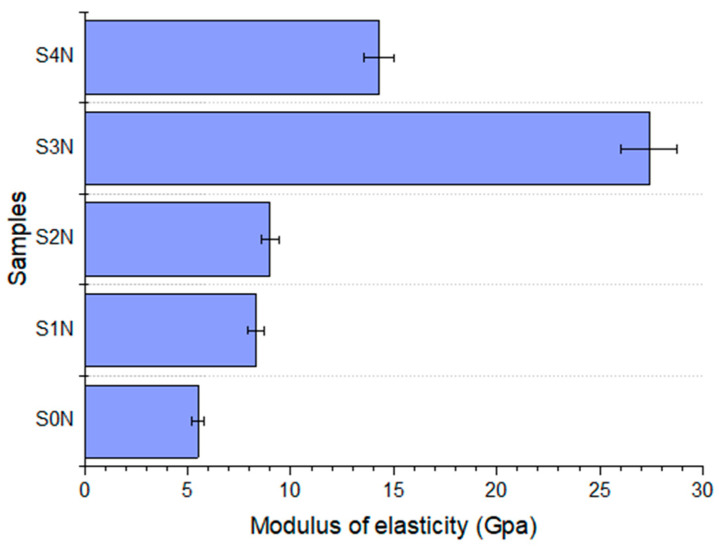
Elastic modulus of Ti-6Al-4V specimens under different UNSM treatment parameters after nitriding.

The greatest improvement in microhardness (over 25%) was achieved under moderate UNSM conditions (amplitude of 20 μm, load of 30 N, RT), which provided an optimal degree of plastic deformation that enhanced nitrogen diffusion. Although increasing the UNSM temperature combined with high loads may further activate diffusion processes, in some cases it leads to reduced hardening efficiency, likely due to recrystallization or the partial relaxation of internal stresses [13,23]. Preliminary UNSM treatment prior to nitriding contributes to an increase in both the elastic modulus and microhardness due to the activation of diffusion processes and the formation of a nanostructured subsurface layer. Repeated high-frequency impacts induce intense plastic deformation, increase dislocation density, and generate crystal lattice defects (dislocations, sub-boundaries, vacancies), which create internal resistance to deformation and thus enhance resistance to elastic strain. This phenomenon, known as dislocation hardening, effectively increases the apparent elastic modulus during surface-sensitive localized measurements [17]. In addition, the presence of a greater number of high-angle grain boundaries and the formation of nanostructures further restrict atomic movement, contributing to localized stiffness enhancement [24]. The experimental results obtained in this study confirm this effect. As shown in Figure 9, the maximum values of microhardness and elastic modulus were achieved under UNSM parameters that provided controlled plastic deformation without overloading the surface (amplitude of 20 μm, load of 30 N, RT). Such treatment promotes a uniform distribution of residual stresses and the formation of a structurally active surface favorable for nitride phase formation during subsequent ion nitriding. Thus, a clear correlation was established between the parameters of the preliminary treatment and the mechanical properties of the modified layer, enabling the targeted design of surface structure and properties for the development of wear-resistant functional coatings [25]. The microhardness values obtained in this study (e.g., up to 25% increase after UNSM + IPN treatment) demonstrate a noticeable improvement compared to those reported for Ti-6Al-4V alloys treated using conventional PVD, plasma ion implantation, and laser nitriding methods, where hardness enhancement typically ranges from 10% to 15% [22]. This suggests that the pre-treatment by UNSM significantly enhances nitrogen diffusion and contributes to the formation of a more hardened surface layer.

To highlight the advantages of the hybrid UNSM-assisted nitriding method developed in this study, Table 4 provides a direct comparison of the obtained results with the selected literature data. It summarizes key mechanical and tribological properties such as surface hardness, elastic modulus, and wear performance. The findings show that the present method achieves comparable or superior results, supporting its effectiveness for surface engineering applications.

## 4. Conclusions

The pre-treatment of Ti-6Al-4V titanium alloy by UNSM prior to IPN leads to significant changes in the structure and properties of the nitrided layer. It was found that varying the UNSM parameters significantly affects the tribo-mechanical characteristics of the surface. UNSM pre-treatment increases microhardness by up to 25% and the elastic modulus by 18% compared to samples without surface activation. Furthermore, UNSM enhances the tribological performance of the nitrided layer. The best results were obtained for the sample treated at A = 30 µm, F = 60 N, T = 400 °C, demonstrating a stable coefficient of friction of approximately 0.55. SEM and XRD analyses confirmed that UNSM promotes the formation of a more uniform and dense nitride layer. Thus, UNSM is shown to be an effective method for improving the structural and functional performance of nitrided layers on Ti-6Al-4V alloy. Owing to the enhanced surface hardness, refined microstructure, and potential for improved wear performance, the surface-treated Ti-6Al-4V alloy is expected to find application in advanced industries such as biomedical engineering (e.g., hip implants, dental screws), aerospace systems, and precision mechanical components exposed to high contact stresses and corrosive environments.

## Figures and Tables

**Table 1:** Chemical composition of Ti-6Al-4V alloy (wt.%).

Source	Method	Key Parameters	Surface Hardness (HV)	Elastic Modulus (GPa)	Wear/Corrosion/Fatigue Resistance	Key Findings
Aringozhina et al. (2025) [20]	EA-UNSM	Amplitude 30 µm, Load 40–60 N	394 → 475 (+20%)	~156	↑ fatigue strength (~8%), ↑ wear resistance	Grain refinement, improved mechanical properties
Zha et al. (2024) [19]	Ultrasonic Surface Rolling	32 kHz, Load ~400 N	325 → 451	124 → 139	Better dynamic mechanical behavior	Nanocrystallization, work hardening
Wang et al. (2021) [8]	Shot Peening + Gas Nitriding	Pre-shot peening, 700–800 °C	Up to 1100 (on steel)	—	↑ wear resistance	Shot peening aids nitriding depth
Zhang et al. (2023) [4]	HCPSN Nitriding	510 °C, 1–4 h	—	—	↑ corrosion resistance (Hank’s solution)	TiN/Ti2N + α-Ti(N), stable in solution
Ongtrakulkij et al. (2022) [26]	Plasma Ion Nitriding	750–800 °C, 5–10 h	Up to 643	—	↑ compressive stress, ↑ fatigue resistance	Dense nitrided layer, better stability
Hsu et al. (2023) [27]	CAD-PVD (TiN/CrN Multilayer)	Bias −150 V	3–5× increase	—	Excellent wear and corrosion resistance	Multilayers enhance protective properties

**Table 2:** Chemical composition of Ti-6Al-4V alloy (wt.%).

Element	Ti	Al	V	Fe	O	C	N	H
Content (wt.%)	bal.	5.5–6.75	3.5–4.5	≤0.30	≤0.20	≤0.08	≤0.05	≤0.015

**Table 3:** Processing conditions for Ti-6Al-4V alloy specimens.

Samples	Sample Designation	Processing Conditions
S0	initial	initial
S1	UNSM-only	UNSM (20 μm, 30 N, RT)
S2	UNSM-only	UNSM (30 μm, 30 N, RT)
S3	UNSM-only	UNSM (30 μm, 50 N, 400 °C)
S4	UNSM-only	UNSM (30 μm, 60 N, 400 °C)
S0N	nitrided	nitrided
S1N	Combined	S1+ nitrided
S2N	Combined	S2+ nitrided
S3N	Combined	S3+ nitrided
S4N	Combined	S4+ nitrided

**Table 4:** Comparative summary of present results and literature.

Source	Method	Surface Hardness (HV)	Elastic Modulus (GPa)	Wear Resistance	Key Findings
This work (S4N)	UNSM + IPN	475 HV	156 GPa	High	Stable friction (0.55), dense TiN layer
Aringozhina et al. [20]	EA-UNSM	475 HV	156 GPa	Improved	Fatigue ↑8%, grain refinement
Zha et al. (2024) [19]	USRP	451 HV	139 GPa	Improved	Nanocrystallization
Wang et al. (2021) [8]	Shot Peening + Gas Nitriding	1100 HV (on steel)	—	Improved	Shot peening aids nitriding depth
Zhang et al. (2023) [4]	HCPSN Nitriding	—	—	Corrosion resistance ↑	TiN/Ti_2_N + α-Ti(N), stable
Hsu et al. (2023) [27]	TiN/CrN Multilayer PVD	3–5× increase	—	Excellent	Multilayers improve protection

## Data Availability

No new data were created or analyzed in this study. Data sharing is not applicable to this article.
